# Dr. William Chace

**Published:** 1892-01

**Authors:** 


Dr. William Chace, of Mayville, Chautauqua County, died at his residence on the morning of Sunday, December 27th, aged about sixty years. For a third of a century Dr. Chace had been prominent in his profession in Western New York, attaining to an eminence far in advance of many in the region of his practice. He was a member of the several medical organizations of his County and State, and also curator of the medical department in the University of Buffalo.

He leaves a widow and four sons, who receive the sympathy of friends and neighbors, as well as that of a liberal profession, in their sudden and trying bereavement.

